# Timing of dialysis initiation in AKI in ICU: international survey

**DOI:** 10.1186/cc11906

**Published:** 2012-12-19

**Authors:** Charuhas V Thakar, James Rousseau, Anthony C Leonard

**Affiliations:** 1 Department of Internal Medicine, University of Cincinnati, 231 Albert Sabin Way, Cincinnati, OH 45267, USA

## Abstract

**Introduction:**

Initiating dialysis in acute kidney injury (AKI) in an intensive care unit (ICU) remains a subjective clinical decision. We examined factors and practice patterns that influence early initiation of dialysis in ICU patients with acute kidney injury.

**Methods:**

An online survey presented nephrologists (international) with three case scenarios with unstated predicted mortality rates of < 10%, 10 - 30% and > 30%. For each case the respondents were asked 4 questions about influences on the decision whether or not to initiate dialysis within 24 hours: Q1, likelihood of initiating dialysis; Q2, threshold of BUN levels (< 50, 50 - 75, 76 - 100, > 100 mg/dl) considered relevant to this decision; Q3, magnitude of creatinine elevation (two to three-fold increase; greater than threefold increase; absolute level > 5 mg/dl regardless of change) considered relevant; Q4, a rank order of the influence of five parameters (BUN level, change of creatinine from baseline, oxygen saturation, potassium level, and urine output), 1 being the most influential and 5 being the least influential.

**Results:**

One hundred seventy-two nephrologists (73% in practice for > 5 years; 70% from the U.S.A.) responded to the survey. The proportion of subjects likely to initiate early dialysis increased (76% to 94%), as did the predicted mortality (p < 0.001). The proportion of subjects considering early dialysis at a BUN level ≤ 75 increased from 17% to 30 to 40% as the predicted mortality of the cases increased (p < 0.0001). The proportion of subjects choosing absolute creatinine level to be more influential than relative increment, went from 60% to 54% to 43% as predicted mortality increased (p < 0.0001). Rank-order analysis indicated that influence of oxygen saturation and potassium level on dialysis decision showed a significant change with severity of illness, but BUN level and creatinine elevation remained less influential, and did not change with severity.

**Conclusions:**

Severely ill patients were more likely to be subjected to early dialysis initiation, but its utility is not clear. Rank-order analysis indicates dialysis initiation is still influenced by "imminent" indications rather than a "proactive" decision based on the severity of AKI or azotemia.

## Introduction

Acute kidney injury (AKI) requiring dialysis is a serious complication in patients admitted to intensive care units (ICUs) and is associated with increased morbidity, mortality, and costs of care [1-3]. The timing of the initiation of dialysis following AKI in the ICU is a subjective clinical decision. By using lower level of azotemia as one of the surrogates for early dialysis, observational studies have shown that timing of initiation may be associated with a survival benefit [4-8]. The recently published clinical practice guidelines for AKI - Kidney Disease: Improving Global Outcomes - propose a stage-based management of AKI, in which consideration of dialysis is recommended in patients experiencing stage II/stage III AKI (AKI stages based on the Acute Kidney Injury Network classification). In practice, however, indications for dialysis in AKI are usually based on medically untreatable complications, including azotemia, hyperkalemia, oliguria/volume overload usually leading to hypoxemia, and severe metabolic acidosis [9]. Additionally, decision of dialysis initiation varies with the overall clinical severity of illness of the patient [10]. Even though the timing of dialysis initiation in AKI has been a subject of clinical interest and a potential area for prospective investigation [11], physician practice preferences and perceptions about early dialysis initiation in AKI are not well understood. Surveys regarding renal replacement therapy in AKI have focused largely on either availability or preferences of different modalities of acute dialysis in the ICU [12-14]. There remains a gap in our knowledge regarding how physicians use the clinical information while making their decision regarding timing of dialysis initiation.

We conducted an international survey of nephrologists about factors that may affect early initiation of dialysis in ICU patients with AKI. We assessed whether differences in clinical severity of illness across different case scenarios would influence physicians' interpretation of objective biochemical information. We examined whether certain biochemical indicators are more influential than others in making the determination of dialysis initiation. We also surveyed physicians about their opinions and perceptions about the potential benefit versus harm of early dialysis in the ICU.

## Materials and methods

An online survey was created and distributed to nephrologists practicing in the US, Canada, or other countries. The nephrologists were asked questions concerning the early initiation of dialytic intervention in patients with AKI in the ICU and the influence on those decisions of various biochemical parameters. Three clinical case scenarios were created (cases A, B, and C); the clinical and laboratory characteristics of the cases were designed such that the predicted hospital mortality rates for cases A, B, and C were less than 10%, 10% to 30%, and greater than 30%, respectively. APACHE III (acute physiology and chronic health evaluation III) criteria were used to determine predicted mortality after considering the clinical and laboratory characteristics in each case scenario.

The description of the cases included detailed clinical and laboratory information. For all three cases, we provided baseline values of blood urea nitrogen (BUN) and serum creatinine. We also provided information on the following laboratory parameters (as recorded on the day of renal consult): level of BUN, serum creatinine level, serum potassium level, and urine output in the previous 24 hours. Of note, the baseline values were similar across all three cases, as were values on the day of consult. Thus, the differences across these cases were based primarily on severity of illness (predicted mortality). This allowed us to determine how the physicians may use the biochemical information in the context of the clinical condition of the patient.

The survey included questions on four areas: Q1 - subjective likelihood of initiating dialysis within 24 hours for each case scenario. Dialysis was considered as 'early initiation' if, given the clinical case scenario, the respondent would initiate the therapy within 24 hours; Q2 - threshold of BUN levels (less than 50, 50 to 75, 76 to 100, and greater than 100 mg/dL) considered in making this decision; Q3 - magnitude of creatinine elevation relative to baseline (two- to threefold increase; greater than threefold increase; absolute level greater than 5 mg/dL regardless of change) considered important; Q4 - a rank ordering of five biochemical parameters in order of their importance to the decision to initiate dialysis in each of the case scenarios (BUN level, change of creatinine from baseline, oxygen saturation, potassium level, and urine output), 1 being the most influential and 5 being the least influential. Toward the end of the survey, we asked questions that were related to characteristics of survey respondents, their practice settings, their training/experience, and their perceptions/practices about early dialysis in AKI in the ICU.

The survey was developed in collaboration with experts from our local institution. We planned the survey around the focus of presenting explicit case scenarios with different severity of illness. The University of Cincinnati Institutional Review Board approved the survey document and the study. Disseminating it to the nephrologists within our local institution, we confirmed the face validity of the survey. The steps of extraction and interpretation were refined internally, prior to external dissemination, and demonstrated that there was 100% re-test validity to the answers. To distribute the survey, we used the following avenues: (a) Nephrology Now web-based portal, (b) the renal field advisory committee of the Veterans Health Administration, (c) the training program directors at academic institutions, and (d) publicly available contact information for nephrologists (local/regional/state). The survey was activated online in fall of 2010, and after few automatic reminders, it was closed in spring of 2011. Data extraction of response and analysis was conducted after the survey was closed to new respondents.

### Statistical analysis

The survey questionnaire was developed by using an online tool, which had been tested internally, within our institution, to ensure the clarity and validity of the questions asked (see Additional file 1 for the survey document). We calculated and reported the frequencies of responses to each of the four questions by all 172 respondents. Significance tests were conducted by comparing case responses to the most severe and least severe case and using a reduced sample of 119 respondents who had addressed all 24 items on the survey. For these comparisons, the response options were collapsed into dichotomies: for the first question, very likely and somewhat likely (likely) versus somewhat unlikely and very unlikely (unlikely); for the second question (regarding BUN threshold), less than 75 mg/dL versus greater than or equal to 75 mg/dL; for the third question, relative increment in creatinine versus absolute level of greater than 5 mg/dL. McNemar's test was used to compare the proportion of respondents answering in each direction on each of the three questions. For the fourth question, we calculated mean rankings for each parameter for the respective case scenarios (1 being most important and 5 being the least important), and compared the scenarios by using a paired *t *test. We also compared, by chi-square test, the characteristics of respondents on the basis of whether they answered the first question as likely (somewhat and very likely combined) or unlikely (somewhat and very unlikely combined) to initiate early dialysis. A two-tailed *P *value of less than 0.05 was considered significant.

## Results

Overall, 172 nephrologists, two of whom were trained in critical care, responded to the survey. Characteristics of those who responded to the survey are shown in Table 1. Seventy-six percent of the respondent sample was male, and 53% had been in practice for more than 10 years. Fifty-eight percent of respondents were based in academic medical centers, and 67% practiced at locations with renal fellowship programs. The majority of respondents (75%) reported that they spent more than 50% of their time in clinical activities and that greater than 25% of their consults originated in the ICU. Fifty-three percent of respondents indicated that, based on the current level of evidence, there is no benefit for initiating early dialysis in AKI; moreover, 35% of respondents indicated that, based on the present evidence, they believed that the risk of early dialysis initiation outweighs the benefit. However, 46% of respondents indicated that they frequently initiate 'early dialysis' in an ICU patient with AKI; of those who perceived that risk of early dialysis outweighed benefit, 42.5% responded that they frequently initiate early dialysis. Seventy-three percent of respondents indicated that they would not have any reservations enrolling patients in a clinical trial that examined early versus late initiation of dialysis in AKI.

**Table 1:** Characteristics of survey respondents

Characteristic	Groups	Proportion of respondents
Gender	Male	76%

Age, years	≤ 40	41%

	41-60	45%

	> 60	14%

Years in practice	< 10	47%

	11-20	20%

	> 20	33%

Type of practice	Academic	58%

	Community-based	22%

	Both	20%

Fellowship program at practice location	Present	67%

Percentage of consults in intensive care unit	< 25%	30%

	26%-50%	60%

	> 50%	10%

Proportion of time devoted to clinical activities	< 50%	25%

	51%-75%	32%

	> 75%	43%

Country of practice	US	68%

	Other	32%

We compared the characteristics of respondents who answered the first question as likely (very likely or somewhat likely combined) for initiating early dialysis with those who responded unlikely (somewhat unlikely or very unlikely combined) for each of the three cases. The only statistically significant difference was found to be the country of practice; those who noted their practicing location to be the US responded that they were less likely to initiate early dialysis as compared with those in practice outside of the US (*P *= 0.041).

**Figure 1:**
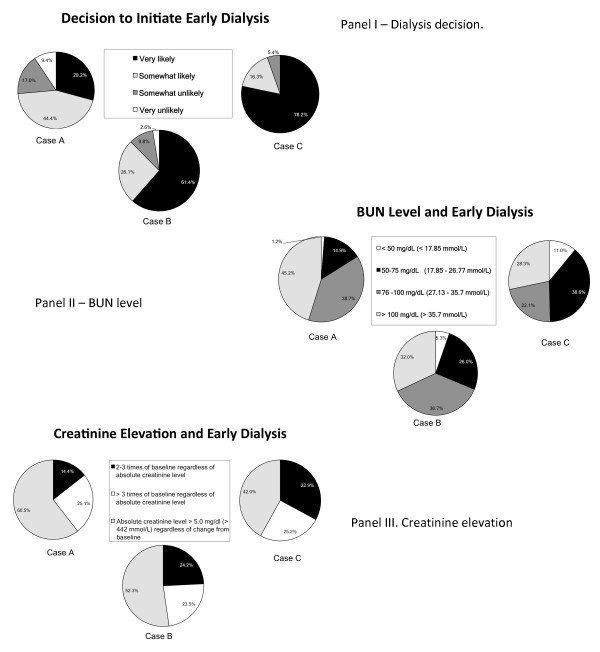
**Differing thresholds of dialysis initiation in acute kidney injury by case severity in the intensive care unit**. Data are from all 172 respondents; 119 out of 172 respondents answered all survey questions. BUN, blood urea nitrogen.

As the severity of illness/predicted mortality of the case scenario increased, the proportion of respondents very likely to initiate early dialysis went up and the threshold of BUN level at which dialysis would have been initiated went down. With regard to creatinine elevation, the majority of the respondents relied on absolute creatinine level rather than relative increment in creatinine as the major indicator to initiate dialysis in the two less severe cases. Even in case C, which had the highest severity of illness, close to half of the respondents still chose absolute creatinine level to be important in initiating early dialysis. The overall trend in responses for all of the 172 respondents is shown in Figure 1.

For the 119 respondents who answered all 24 items on the survey, the proportion of subjects saying they would be likely (very likely or somewhat likely) to initiate early dialysis increased from 76% in the least severe case to 94% in the most severe case (*P *< 0.0001). The proportion of subjects considering early dialysis at a BUN level of not more than 75 mg/dL also increased from 17% in the least severe case to 40% in the most severe (*P *< 0.0001). The proportion of subjects choosing absolute creatinine level over the relative increment in creatinine in their decision to initiate early dialysis went from 60% in the least severe case to 43% in the most severe (*P *< 0.0001).

**Figure 2:**
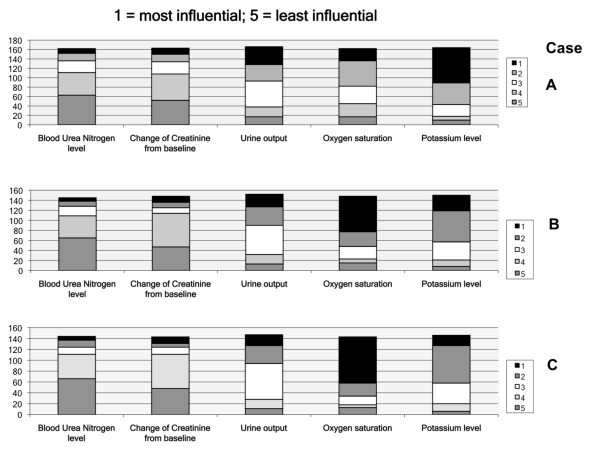
**Rank order of biochemical parameters that influence dialysis decision**. Data are from all 172 respondents.

The results of the rankings of the influence of the five biochemical parameters are shown in Figure 2 (1 is most influential and 5 is least). Generally, oxygen saturation and potassium levels remained predominantly influential parameters in initiating dialysis across all scenarios. In Table 2, we show the average rank order based on the responses for each of the biochemical parameters, for each of the three cases. The mean ranking for oxygen saturation went down (became more influential) and potassium level went up (became less influential) as predicted mortality increased (paired *t *tests *P *< 0.0001 for each). In other words, potassium levels had the most influence on the dialysis decision in the case with lowest severity of illness, whereas oxygen level was most influential in the case with highest severity of illness. Mean rankings for urine output, BUN level, and creatinine elevation did not significantly differ across the least severe and the most severe cases.

**Table 2:** Survey responses for early initiation of dialysis in acute kidney injury in the intensive care unit

Questions	Qualifiers	Case A	Case B	Case C
Consider initiating early dialysis	Likely	91 (76%)	107 (90)	112 (94%)

	Unlikely	28 (24%)	12 (10%)	7 (6%)

Urea level, number (percentage)	< 50 mg/dL	1 (1)	6 (5)	13 (11)

	50-75 mg/dL	19 (16)	30 (25)	46 (39)

	76-100 mg/dL	48 (40)	46 (39)	28 (24)

	> 100 mg/dL	51 (43)	37 (31)	32 (27)

Creatinine elevation	2-3 times baseline	15 (13)	28 (24)	37 (31)

	> 3 times baseline	32 (27)	27 (23)	31 (26)

	> 5.0 mg/dL	72 (60)	64 (54)	51 (43)

Biochemical parameters^a^	Blood urea nitrogen	3.9 (1.2)	4.0 (1.1)	4.1 (1.1)

	Creatinine	3.8 (1.3)	3.9 (1.2)	3.9 (1.2)

	Urine output	2.7 (1.2)	2.7 (1.1)	2.7 (1.0)

	Oxygen	2.6 (1.2)	2.1 (1.3)	1.8 (1.3)

	Potassium	1.9 (1.2)	2.3 (1.1)	2.5 (1.0)

Results include the 119 respondents (out of 172) who answered all of the questions in the survey. ^a ^Ranking (mean with standard deviation) for each biochemical was averaged for each parameter; 1 was most influential and 5 was least influential.

## Discussion

We report the results of a survey of nephrologists regarding their practice of and perceptions about initiating early dialysis in patients with AKI in the ICU. The survey results indicate that physicians tend to decide on early dialysis for the more severely ill patients in the ICU. The results also suggest that the decision for dialysis initiation is influenced more heavily by complications of AKI - that is, hyperkalemia, volume overload, and so on - than by measures of severity of kidney injury.

Very few studies have examined survey questionnaires focused on issues arising from AKI. Ricci and colleagues [14] analyzed a survey given to attendees of a nephrology conference in Vicenza, Italy, to study practice patterns in the management of AKI in critically ill patients. The survey found that practitioners used multiple definitions of AKI. Over 90% of respondents used continuous renal replacement therapy (CRRT) as one of the modalities in the ICU; however, the treatment prescription showed significant differences among specialists. The most commonly prescribed dose was 35 mL/kg per hour, although this survey pre-dated the most recent clinical trials examining the optimal dose of dialysis in AKI. Of the participants, 90% said that they used RRT for non-renal indications, and 60% admitted the lack of scientific evidence as a limiting factor in its use.

In another study, Overberger and colleagues [13] surveyed clinical trial sites before patient enrollment in a Veterans Affairs/National Institutes of Health trial to assess patterns of clinical practice of RRT use in patients with AKI. Intermittent hemodialysis (IHD) and CRRT were the most commonly used modalities of therapy. IHD was most commonly provided on a thrice-weekly schedule, with only infrequent assessment of the delivered dosage of therapy. Most practitioners reported that they did not dose CRRT on the basis of patient weight, yet the average prescribed dosage of therapy corresponded to a weight-based dosage of 20 to 25 mL/kg per hour. In another survey, Hyman and Mendelssohn [12] surveyed (by mail questionnaire) all adult academic and community-registered Canadian nephrology centers about approaches to dialysis in AKI. Comparing contemporary dialysis methods with those of 5 years ago, they reported the largest increase was in CRRT (26% versus 9%). Both IHD and peritoneal dialysis as modalities for AKI decreased in utilization. In 2002, the predominant CRRT methods used venovenous access (80%), whereas 5 years earlier arteriovenous was the most common (52%). The survey found that, notwithstanding a lack of definitive evidence of superior outcomes with CRRT compared with other modalities, the utilization of CRRT dramatically increased for the treatment of AKI in Canada. More recently, in a study of well-conducted surveys, Clark and colleagues [15] examined the factors that would prompt respondents to initiate RRT for AKI. This study indicated that potassium level and pulmonary edema were the most common triggers for prompt initiation, and over 90% of respondents favored a prospective clinical trial to determine optimal timing [15].

Our study contrasts with these prior reports in that we studied the question of timing of dialysis initiation rather than utilization of a specific modality. First, we designed a unique survey focusing on specific case scenarios but did not provide an explicit measure of severity of illness or predicted mortality - this was left for the survey respondent to consider in their decision making, as they answered the questions. Thus, we were able to assess physician propensities to initiate early dialysis based on each case scenario. The data indicate that a greater proportion of respondents were likely to initiate early dialysis in patients with higher severity of illness. Second, across all three case scenarios, we provided survey respondents with the same biochemical parameters that are usually considered when making decisions regarding initiating dialysis. In this part of the survey, we observed that the BUN level at which dialysis would be initiated was lower in patients who were more severely ill. Interestingly, the majority of respondents assigned greater importance to the absolute level of creatinine (> 5 mg/dL) than to a relative elevation of creatinine as the factor affecting their decision to initiate dialysis. Finally, for each case scenario, we asked nephrologists to rank the clinical parameters on their degree of influence on the decision to initiate early dialysis. Hyperkalemia and hypoxemia took precedence over indicators of azotemia in influencing dialysis decisions across the spectrum of illness severities. Hyperkalemia was most influential in deciding dialysis initiation in the less severely ill case, whereas hypoxemia was most influential in the high severity of illness case.

This suggests that patients who are more severely ill are more likely to be subjected to the intervention of 'early dialysis'. It is speculated that these patients are unlikely to experience a survival benefit despite such an intervention. The present study also indicates that the decision to initiate dialysis is most influenced by parameters that represent more urgent indications to initiate dialysis rather than a proactive decision based on the degree of kidney injury or anticipation of therapy and that the association between the timing of dialysis initiation and factors related to non-renal organ dysfunction (for example, volume overload leading to pulmonary edema) needs more detailed investigation.

As an additional note, since APACHE III may not accurately predict mortality in subjects with AKI and AKI-specific predictive models consistently show a reverse relationship between serum creatinine level at dialysis initiation and outcome (for example, because of malnutrition, dilution, short duration from insult to renal complications, or severe critical illness), we assessed parameters in our clinical scenarios to predict 60-day mortality on the basis of the ATN (Acute Renal Failure Trial Network) study [16]. We observed that, had they been initiated on dialysis on the day of laboratory data provided, the first two case scenarios would have had a less than 10% predicted mortality but that the third case would have had a greater than 30% predicted mortality.

Our survey respondents represented a broad spectrum of nephrologists from the US and from other countries, a wide distribution of years in practice, and a variety of practice settings. Notably, 53% of respondents indicated that, based on the current level of evidence, there is no benefit in initiating early dialysis in AKI; moreover, 35% of respondents indicated that, based on the present evidence, they believed that the risk of early dialysis initiation outweighs the benefit. However, 46% of respondents indicated that they frequently initiate 'early dialysis' in an ICU patient with AKI, and 73% of them indicated that they would not have any reservation in enrolling patients in a clinical trial that examined early versus late initiation of dialysis in AKI. This shows that a lack of evidence or clear consensus is partly responsible for the inconsistencies in the perceptions and practice with regard to timing of dialysis initiation; on the other hand, it may suggest that this decision is complex and is associated with a reasonable degree of subjectivity.

There are limitations to our analysis. The sample is not large, and the mode of distributing our survey may have led to a selection bias in potential responders. However, considering the self-identified characteristics of the respondents, we believe that the sample is representative of the spectrum of practicing nephrologists, primarily within the US. Our survey was taken only once by each respondent; thus, we cannot say whether the same respondent would have consistently chosen the same responses.

## Conclusions

Survey respondents were more likely to initiate early dialysis in those individuals who are more severely ill; but its beneficial effects in these patients are unclear. Parameters most influential in determining dialysis initiation were complications of AKI, such as hyperkalemia and hypoxemia due to volume overload, whereas the degree of severity of kidney injury or markers of azotemia played a less important role in the early dialysis decision. About half of the respondents said that the current evidence to support the benefits of early dialysis is weak, and 1 in 3 respondents thought that early dialysis might actually be harmful. Based on the survey, we conclude that the timing of dialysis initiation remains a clinical decision, one that is affected more by subjective assessment of severity of illness than objectively measured parameters of azotemia; its true impact on patient outcomes needs prospective investigation.

## Key messages

• Patients who are more severely ill are likely to be subjected to early initiation of dialysis in AKI in the ICU, but the utility of this practice remains unproven.

• Parameters of azotemia at which dialysis is initiated change with severity of illness.

• The BUN level at which dialysis is initiated depends on the severity of illness of the patient; absolute level of creatinine is more influential than relative increase in serum creatinine.

• Rank-order analysis indicates that dialysis initiation may be determined by 'imminent' indications rather than a proactive decision based on the degree of azotemia.

• Over half of the survey respondents indicated that the evidence to support early initiation of dialysis is weak, and 1 in 3 respondents believed that initiating early dialysis may be harmful.

## Abbreviations

AKI: acute kidney injury; APACHE: acute physiology and chronic health evaluation; BUN: blood urea nitrogen; CRRT: continuous renal replacement therapy: ICU: intensive care unit; IHD: intermittent hemodialysis; Q: question; RRT: renal replacement therapy.

## Competing interests

The authors declare that they have no competing interests.

## Authors' contributions

CVT designed the study, assisted in designing the survey, collated the responses, assisted in data analysis, and prepared the manuscript. JR designed and conducted the online survey, obtained institutional review board and regulatory approvals, and contributed in writing and editing the manuscript. ACL conducted statistical analysis and contributed in writing and editing the manuscript. All authors read and approved the final manuscript.

## Supplementary Material

Additional file 1 **Survey questionnaire**. The survey questionnaire, including the case scenarios, that were distributed. Click here for file
