# Supplementary material for: Timing of dialysis initiation in AKI in ICU: international survey
Source: Crit Care. 2012 Dec 19;16(6):R237. doi: 10.1186/cc11906 (PMC3672626; doi:10.1186/cc11906)
Supplement: Additional file 1 — Survey questionnaire. The survey questionnaire, including the case scenarios, that were distributed. [file cc11906-S1.PDF]

Factors Affecting Timing of Dialysis Initiation in Acute Kidney

1. Survey Introduction

The purpose of this research survey is to study the factors and that may affect early initiation of dialysis in acute kidney injury (AKI) patients in Intensive Care Unit (ICU) settings.

This is a research project being conducted by investigators from the Division of Nephrology and Hypertension and Department of Internal Medicine at the University of Cincinnati. You are invited to participate in this research project because you are either a nephrologist or a healthcare provider in critical care setting. Your participation in this research study is voluntary, and appreciated.

The procedure involves completing an online survey that will take approximately 15 minutes. The survey consists of three different case scenarios, representative of ICU patients with AKI, with a set of associated questions. Your responses will be kept confidential, and no identifiable information is being collected.

The study is approved by the Institutional Review Board of the University of Cincinnati. If you have any questions about this survey please contact Dr. Charuhas V. Thakar (charuhas.thakar@uc.edu) or Dr. James Rousseau (james_rousseau@hotmail.com).

This research has been reviewed according to SurveyMonkey University IRB procedures for research involving human subjects.

2. Case Scenario

1. Nephrology consultation is requested on a 66 year old man with a history of coronary artery disease and hypertension. Two days ago he had onset of abdominal pain, low-grade fever, myalgias, and nausea that has persisted. Ten days ago, he underwent a cardiac cathertization for unstable angina followed by stent placement for a 80% right coronary artery lesion.

On physical exam today, temperature was 100.6° F and Blood pressure is 140/96, Respiratory rate 18, pulse 85, and Oxygen saturation 97% on 2 L oxygen.

On cardiac examination, a left carotid bruit is present. Pulmonary examination reveals lungs clear to auscultation. There is trace edema and the distal pulses are faint but present. A netlike violaceous rash is visible over the legs and the left toe is cool and cyanotic.

Laboratory studies: Metric Units (SI Units)

Hemoglobin 9.0 g/dL (90 g/L)
WBC 6700/uL (6.9 x 10*9/L)
Platelets 434, 000/uL (434 x 10*9/L)
Sodium 137 meq/L (137 mmol/L)
Potassium 5.2 meq/L (5.2 mmol/L)
Chloride 105 meq/L (105 mmol/L)
Bicarbonate 20 meq/L (20 mmol/L)
BUN 60 mg/dL (21.4 mmol/L) [baseline was 12mg/dL (4.28 mmol/L)]
Creatinine 4.5 mg/dL (397.8 mmol/L) [baseline was 1.4 mg/dL (123.8 mmol/L)]
24 hour urine output 600 cc (10 - 20 ml/hour for last 8 hours)

Urinary indices are consistent with intrinsic kidney injury.

1. Given the above parameters, how likely is it that you would initiate dialysis within the next 24 hrs?

- Very likely

- Somewhat likely
- Somewhat unlikely
- Very unlikely

2. At what threshold/level of BUN would you consider initiating dialysis within the next 24 hours, given all of the other above parameters being the same?

- < 50 mg/dL (< 17.85 mmol/L)
- 50-75 mg/dL (17.85 - 26.77 mmol/L)
- 76 -100 mg/dL (27.13 - 35.7 mmol/L)
- > 100 mg/dL (> 35.7 mmol/L)

3. At what creatinine level would you consider initiating dialysis within the next 24 hours, given all other above parameters being the same?

- 2-3 times of baseline regardless of absolute creatinine level
- > 3 times of baseline regardless of absolute creatinine level
- Absolute creatinine level > 5.0 mg/dl (> 442 mmol/L) regardless of change from baseline

4. Please rank the following parameters, from 1 to 5, based on how influential they would be in your decision to initiate dialysis within the next 24 hours in this particular patient? (1 being the most influential and 5 being the least influential)

	1	2	3	4	5
Blood Urea Nitrogen level					
Change of Creatinine from baseline					
Oxygen saturation					
Potassium level					
Urine output					

3. Case Scenario

1. A 55-year old woman with a past medical history of mitral valve prolapse and hypertension was recently hospitalized with a diagnosis of Strep Viridans endocarditis and was discharged on home antibiotics with a serum creatinine of 0.8 mg/dL (70.7 mmol/L).

On day 16 of therapy, the patient complained of fatigue, anorexia, and nausea and was readmitted to the hospital upon which a nephrology consult was obtained.

On physical examination on admission, temperature is 36.8 C (98.2 F). Her pulse rate is 115/min, respiratory rate is 26/min, blood pressure is 167/98 mm Hg, and oxygen saturation is 90% on 50% Oxygen.

Mucous membranes are moist, and there is no rash. There is jugular venous distention. Lung examination reveals crackles in lower lung fields bilaterally. There is a soft systolic murmur at the apex. Lower extremity pulses are 1+, with 1+ edema.

Laboratory studies: Metric Units (SI Units)
Hemoglobin 11.6 g/dL (116 g/L)
Leukocyte count 8500/microL (8.5 x 10*9/L)
65% neutrophils
30% lymphocytes
3% monocytes
2% basophils

Platelet count 152,000/microL (152 x 10*9/L)
Blood urea nitrogen 60 mg/dL (21.42 mmol/L) [baseline BUN was 12 mg/dL (4.28 mmol/L)]
Creatinine 4.5 mg/dL (397.8 mmol/L) [baseline creatinine was 0.8 mg/dL (70.7 mmol/L)
Sodium 143 meq/L (143 mmol/L)
Potassium 5.5 meq/L (5.5 mmol/L)
Chloride 109 meq/L (109 mmol/L)
Bicarbonate 18 meq/L (18 mmol/L)

Calcium 8.7 mg/dL (2.175 mmol/L)
Phosphorus 4.7 mg/dL (1.51 mmol/L)
24 hour urine output 600 cc (10 – 20 ml/hour)

Arterial Blood Gas (with patient breathing 50% O_2)
pH 7.25 pO_2 70 mm Hg pCO_2 24 mm Hg

Urinary indices are consistent with intrinsic kidney injury.

Given the above parameters, how likely is it that you would initiate dialysis within the next 24 hrs?

- Very likely
- Somewhat likely
- Somewhat unlikely
- Very unlikely

2. At what threshold/level of BUN would you consider initiating dialysis within the next 24 hours, given all of the other above parameters being the same?

- < 50 mg/dL (17.85 mmol/L)
- 50-75 mg/dL (17.85 - 26.77 mmol/L)
- 76 -100 mg/dL (27.13 - 35.7 mmol/L)
- > 100 mg/dL (> 35.7 mmol/L)

3. At what threshold/level of Creatinine would you consider initiating dialysis within the next 24 hours, given all of the other above parameters being the same?

- 2-3 times baseline regardless of absolute level
- > 3 times baseline regardless of absolute level
- Absolute level > 5.0 mg/dL (442 mmol/L) regardless of change from baseline

4. Please rank the following parameters, from 1 to 5, based on how influential they would be in your decision to initiate dialysis within the next 24 hours in this particular patient? (1 being the most influential and 5 being the least influential)

	1	2	3	4	5
Blood Urea Nitrogen level	○	○	○	○	○
Change of Creatinine from baseline	○	○	○	○	○
Oxygen saturation	○	○	○	○	○
Potassium level	○	○	○	○	○
Urine output	○	○	○	○	○

4. Case Scenario

1. Renal consult is obtained on post-operative day 9 on a 56-year old man status post triple vessel CABG. On post-op day 3 he developed fever, cough, chest pain, and evidence of lower lobe infiltrate on x-ray; nosocomial pneumonia was diagnosed and treated with Vancomycin and Piperacillin/Tazobactam.

On physical examination, the blood pressure is 95/60 mm Hg; pulse rate, 125/min; respiratory rate, 30/min; and temperature 39.0 C (102.2 F). Oxygen saturation on 100% oxygen is 91%.

There is jugular venous distension. Cardiopulmonary examination reveals diffuse crackles. 2+ lower extremity edema is present. Dopamine was initiated for blood pressure support.

Laboratory studies: Metric Units (SI Units)

Hemoglobin 9 g/dL (90 g/L)
Leukocyte count 18,000/microL (18 x 10*9/L)
Platelet count Normal
Sodium 140 meq/L (140 mmol/L)
Potassium 5.5 meq/L (5.5 mmol/L)
Bicarbonate 19 meq/L (19 mmol/L)
Chloride 100 meq/L (100 mmol/L)
Blood urea nitrogen 60 mg/dL (21.42 mmol/L) [Baseline Normal)
Serum creatinine 4.5 mg/dL (397.8 mmol/L) [Baseline 1.0 mg/dL (88.4 mmol/L)
24 hour urine output 600 cc

Arterial blood gasses pH 7.20, PCO2 26 mm Hg
pO2 65% (on 100% oxygen)

Urinary indices are consistent with intrinsic kidney injury.

Given the above parameters, how likely is it that you would initiate dialysis within the next 24 hrs?

- Very likely
- Somewhat likely
- Somewhat unlikely
- Very unlikely

2. At what threshold / level of BUN would you consider initiating dialysis within the next 24 hours, given all of the other above parameters being the same?

- < 50 mg/dL (< 17.85 mmol/L)
- 50-75 mg/dL (17.85 - 26.77 mmol/L)
- 76 -100 mg/dL (27.13 - 35.7 mmol/L)
- > 100 mg/dL (> 35.7 mmol/L)

3. At what threshold / level of Creatinine would you consider initiating dialysis within the next 24 hours, given all of the other above parameters being the same?

- 2-3 times baseline regardless of absolute level
- > 3 times baseline regardless of absolute level
- Absolute level > 5.0 mg/dL (442 mmol/L) regardless of change from baseline

4. Please rank the following parameters, from 1 to 5, based on how influential they would be in your decision to initiate dialysis within the next 24 hours in this particular patient? (1 being the most influential and 5 being the least influential)

	1	2	3	4	5
Blood Urea Nitrogen level					
Change of Creatinine from baseline					
Oxygen saturation					
Potassium level					
Urine output					

5. Practice Patterns

1. Based on current evidence, do you believe initiation of “early dialysis” for ICU patients with acute kidney injury (AKI) is beneficial?

- Yes
- No

2. Based on your current practice patterns, do you frequently initiate early dialysis for an ICU patient with AKI?

- Yes
- No

3. Based on current level of evidence, does the potential risk of early dialysis in ICU patients with AKI outweigh the potential benefit?

- Yes
- No

4. Would you have any reservation enrolling an ICU patient with AKI in a trial that compared early initiation of dialysis versus no treatment?

- Yes
- No

5. Do you consider a patients' severity of illness upon ICU admission in your decision to initiate dialysis in a patient with AKI?

- Yes
- No

6. Please tell us about yourself

1. Sex:

- Male
- Female

2. Age:

- < 30
- 30-40
- 41-50
- 51-60
- >60

3. How many years have been in practice?

- <5
- 6-10
- 11-20
- >20

4. Ethnicity:

- Caucasian
- African American
- Asian/Pacific Islander
- Asian
- Hispanic
- Other

5. What is your primary area of specialty?

- Nephrology
- Critical Care
- Other

6. Your current practice is:

- Academic
- Community-based
- Both

7. Are you a currently enrolled in a training program (student/resident/fellow)?

- Yes
- No

8. Is your practice setting associated with a renal fellowship program?

- Yes
- No

9. While providing inpatient consult services, what is the approximate percentage of overall consults seen within the ICU?

- < 25%
- 26-50%
- >50%

10. Does your institution have either CRRT or SLED available in ICU settings?

- CRRT
- SLED

11. What percent of your time do you spend on clinical activities?

- < 25%
- 26-50%
- 51-75%
- >75%

12. Do you practice in the United States?

- Yes
- No

Thank you for your participation.
